# TFP5 peptide, derived from CDK5-activating cofactor p35, provides neuroprotection in early-stage of adult ischemic stroke

**DOI:** 10.1038/srep40013

**Published:** 2017-01-03

**Authors:** Ya-Bin Ji, Pei-Pei Zhuang, Zhong Ji, Yong-Ming Wu, Yong Gu, Xiao-Ya Gao, Su-Yue Pan, Ya-Fang Hu

**Affiliations:** 1 Department of Neurology, Nanfang Hospital, Southern Medical University, Guangzhou, China; 2 Department of Neurology, Zhujiang Hospital, Southern Medical University, Guangzhou, China

## Abstract

Cyclin-dependent kinase 5 (CDK5) is a multifaceted protein shown to play important roles in the central nervous system. Abundant evidence indicates that CDK5 hyperactivities associated with neuronal apoptosis and death following ischemic stroke. CDK5 activity increases when its cofactor p35 cleaves into p25 during ischemia. Theoretically, inhibition of CDK5/p25 activity or reduction of p25 would be neuroprotective. TFP5, a modified 24-aa peptide (Lys254-Ala277) derived from p35, was found to effectively inhibit CDK5 hyperactivity and improve the outcomes of Alzheimer’s disease and Parkinson’s disease *in vivo*. Here, we showed that intraperitoneal injection of TFP5 significantly decreased the size of ischemia in early-stage of adult ischemic stroke rats. Relative to controls, rats treated with TFP5 displayed reduced excitotoxicity, neuroinflammation, apoptosis, astrocytes damage, and blood-brain barrier disruption. Our findings suggested that TFP5 might serve as a potential therapeutic candidate for acute adult ischemic stroke.

Multiple mechanisms of ischemic stroke have been described^1^, and designing strategies to counter these mechanisms maybe neuroprotective. The cyclin-dependent kinase (CDK) family, especially CDK5, play important roles in excitotoxicity and apoptosis in ischemic neuronal death^2^,^3^,^4^. Normal CDK5 activity in the brain is maintained by its main cofactor, p35^5^. However, in some pathological conditions, such as ischemia, calpain-directed proteolysis can cleave p35 into a N-terminal myristoylated membrane tether and a shorter, more stable form of the cofactor, p25^6^,^7^,^8^. The CDK5/p25 complex is an abnormally hyperactive hyperphosphorylated substrates, thereby leading to neuronal death^9^. Kinase inhibitors that target ATP binding sites in CDKs, such as roscovitine and aminothizole, have been evaluated as potential therapeutic agents^10^,^11^,^12^,^13^. Unfortunately, these compounds can produce serious side effects due to their lack of specificity for CDK5. Previous studies have shown that TFP5, which was a 24-aa peptide (Lys254-Ala277) derived from truncated p35, competed with p25 for binding to CDK5, inhibited CDK5/p25 hyperactivity, and improved Alzheimer’s disease (AD) and Parkinson’s disease (PD) outcomes *in vivo*^14^,^15^. Thus far, strategies to specifically inhibit CDK5 hyperactivity have not been studied in adult ischemic stroke. Based on the similar pathological mechanism of CDK5 hyperactivity in AD, PD, and ischemia, as well as the specific neuroprotection provided by TFP5, we designed this study to determine the efficacy of TFP5 in adult rats with transient middle cerebral artery occlusion (MCAO) by assessing ischemic size, excitotoxicity, neuroinflammation, apoptosis, astrocyte and blood-brain barrier (BBB) status. We postulated that TFP5 is neuroprotective for adult ischemic stroke.

## Results

### TFP5 reduced the infarct size in adult rats with ischemic stroke

**Figure 1:**
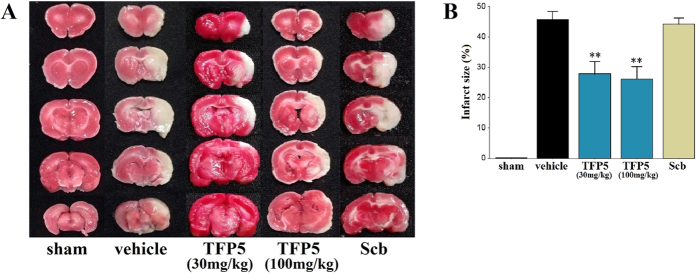
TFP5 reduced the infarct size in animals that underwent 2 h of MCAO and 48 h of blood reperfusion, and no apparent difference between 30 mg/kg and 100 mg/kg TFP5 groups. (**A**) TTC staining showed infarct regions as white regions and non-infarct regions as red regions. (**B**) The bar graph of infarct sizes in all groups. Bars represent means ± SEs. **p < 0.01 versus vehicle group. MCAO, middle cerebral artery occlusion; TTC, 2,3,5-triphenyltetrazolium chloride.

2,3,5-triphenyltetrazolium chloride (TTC) staining was used to confirm and measure infarction lesions located in the MCAO territory. Infarct size was measured in the sham animals (N = 2) and in ischemic animals (Fig. 1A). The ischemic animals were divided into four groups (N = 8/group): a vehicle group, two TFP5 groups (30 mg/kg, 100 mg/kg), and a Scb group (30 mg/kg). Significant differences in infarct size were revealed between the TFP5 groups (30 mg/kg, 27.9 ± 4.0%; 100 mg/kg, 26.1 ± 4.1%) and the non-TFP5 groups (vehicle, 45.7 ± 2.7%; Scb, 44.2 ± 2.0%) (p < 0.01) (Fig. 1B). Infarct size did not differ between the vehicle and Scb groups, nor between the two TFP5 groups (p > 0.05).

### The effect of TFP5 injection on the expression of p25, p35 and CDK5 in ischemic brain tissue

**Figure 2:**
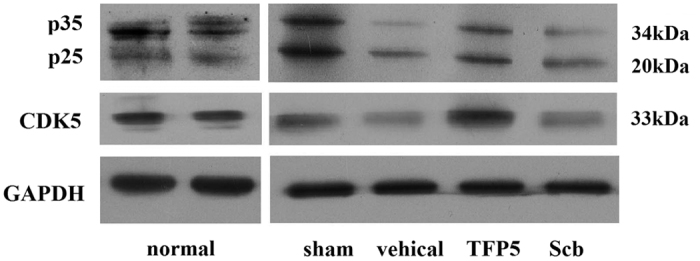
The effect of TFP5 for the level of p25, p35 and CDK5 in the whole ischemic hemispheres with 2-mm-thick coronal slices of rats. In normal rats, p35 expressions were apparently higher than p25. In other all animals, p35 expressions were close or apparently lower than p25, even in sham group, which might be induced by the stress during surgeries. The expressions of p25, p35 and CDK5 in vehicle and Scb animals were lower than sham and TFP5 animals, which indicated the non-ischemic tissues in ischemic hemispheres of animals with TFP5 injection were more than vehicle and Scb animals. Data was from four independent experiments. CDK5, cyclin-dependent kinase 5; GAPDH, glyceraldehyde-3-phosphate dehydrogenase.

Previous studies have showed that the CDK5/p25 hyperactivity could be suppressed specifically by TFP5. Here we observed the ratio of p25/p35 and CDK5 expression in ischemic hemispheres by western blot (Fig. 2). Data showed that the p25/p35 ratio was lower in the TFP5 group than in the control groups, which was disturbed by the possible stress during surgeries. The stress was suggested by the higher expression of p25 in animals with surgeries than normal animals. The CDK5 expression in animals with TFP5 injection was higher than control animals, which indicated that the non-ischemic tissues of animals with TFP5 injection were more than control animals, which was coincident with the data of TTC staining.

### The phosphorylated N-methyl-D-aspartate (NMDA) receptor subunit 2 A (NR2A) at Ser1232 site (p-NR2A^S1232^) was reduced after TFP5 injection

**Figure 3:**
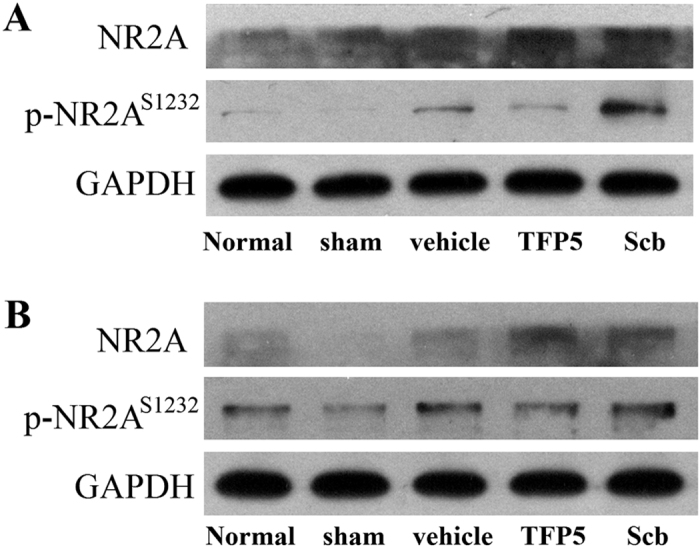
A dose of 30 mg/kg of TFP5 reduced p-NR2A^S1232^ levels both in cortex neighbouring ischemic regions (**A**) and hippocampus of ischemic hemisphere (**B**) at 48 h after 2 h ischemia. The levels of NR2A and p-NR2A^S1232^ in animals with ischemic stroke were higher than normal and sham animals. However, among three ischemic groups, the p-NR2A^S1232^ levels in TFP5 group were lower than vehicle and Scb groups. Data was from four independent experiments. p-NR2A^S1232^, phosphorylated N-methyl-D-aspartate receptor subunit 2A at Ser1232; GAPDH, glyceraldehyde-3-phosphate dehydrogenase.

CDK5 activation during ischemia induces cell death by directly phosphorylating NR2A^S1232^. Here, we wanted to check whether phosphorylation of NR2A^S1232^ would be inhibited by TFP5. NR2A and p-NR2A^S1232^ in cortex neighbouring ischemic regions and hippocampus of ischemic hemisphere were measured. As showed in Fig. 3A and B, levels of NR2A and p-NR2A^S1232^ both in cortex and hippocampus increased at 48 h after 2 h ischemia, while the p-NR2A^S1232^ levels in animals treated with TFP5 were lower than vehicle and Scb groups, which indicated that TFP5 inhibited on phosphorylation of NR2A at Ser1232 site.

### Apoptosis, astrocyte and BBB injuries, and neuroinflammation were alleviated after TFP5 injection

**Figure 4:**
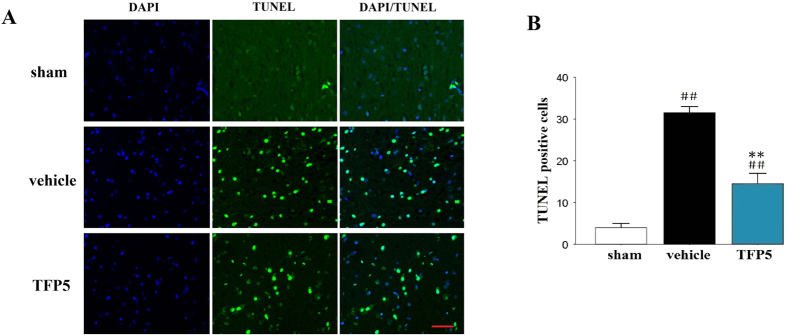
A dose of 30 mg/kg of TFP5 significantly reduced apoptosis in the ischemic regions of animals that underwent 2 h of MCAO and 48 h of blood reperfusion. (**A**), Representative photomicrographs of immunofluorescence labeled with DAPI (blue) and apoptosis of brain cells (green) in the ischemic regions of animals at 48 h after 2 h-MCAO. Bar, 20 μm. (**B**), Semiquantitative results of apoptosis of brain cells. The bars represent means ± SEs. ^##^p < 0.01 versus sham group; **p < 0.01 versus vehicle group. MCAO, middle cerebral artery occlusion; DAPI, 4′,6-diamidino-2-phenylindole.

TUNEL staining demonstrated that the ischemic brains exhibited extensive apoptosis of brain cells. Brain cell loss in animals given 30 mg/kg TFP5 was markedly lower than that in vehicle animals (N = 5/group; p < 0.01; Fig. 4A,B).

**Figure 5:**
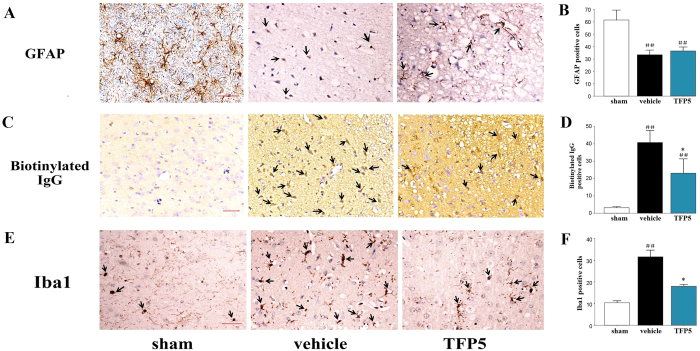
TFP5 at a dose of 30 mg/kg alleviated astrocyte and BBB injuries, as well as neuroinflammation in animals that underwent 2 h of MCAO and 48 h of blood reperfusion. (**A,C,E**), Representative photomicrographs of immunohistochemistry for GFAP, biotinylated IgG, and Iba1 in the ischemic regions at 48 h after 2 h-MCAO (arrows). Bar, 20 μm. (**B,D,F**), Semiquantitative results of GFAP, biotinylated IgG, and Iba1. The bars represent means ± SEs. ^##^p < 0.01 versus sham group. *p < 0.05 versus vehicle group. MCAO, middle cerebral artery occlusion; BBB, blood-brain barrier; GFAP, glial fibrillary acidic protein; Iba1, ionized calcium-binding adapter molecule 1.

To assess the effect of TFP5 on astrocytes in the ischemic regions, we measured the expression of the astrocytes specific marker, glial fibrillary acidic protein (GFAP) in brains. Interestingly, we did not observe activation of astrocytes in the ischemic regions. Instead, the GFAP-positive cells contained less glial filaments in the vehicle group than in the TFP5 group (N = 5/group; Fig. 5A,B), although there was no significant difference between the number of GFAP-positive cells in the vehicle and TFP5 groups (p > 0.05). Our data suggested that TFP5 protected astrocytes in the early-stage of ischemic stroke.

**Figure 6:**
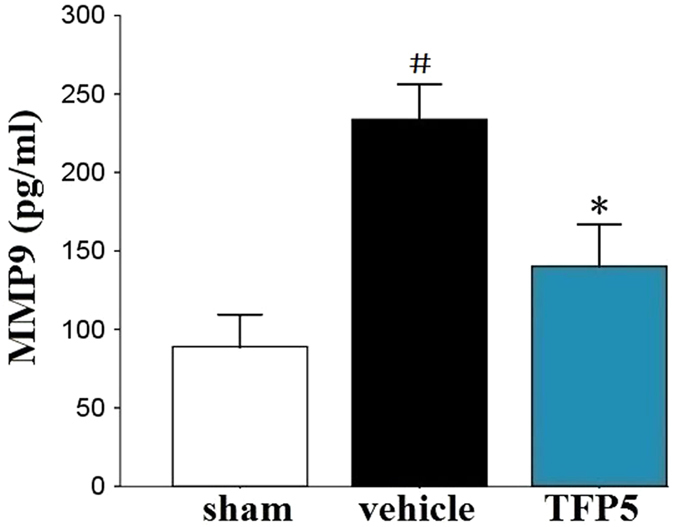
TFP5 at a dose of 30 mg/kg reduced significantly serum MMP9 levels in animals that underwent 2 h of MCAO and 48 h of blood reperfusion. The bars represent means ± SEs. ^#^p < 0.05 versus sham group. *p < 0.05 versus vehicle group. MCAO, middle cerebral artery occlusion; MMP9, matrix metallopeptidase 9.

To determine whether TFP5 can relieve BBB disruption, expression of biotinylated IgG^16^ in the brain ischemic regions and serum matrix metallopeptidase 9 (MMP9) levels^17^,^18^ were determined by immunohistochemistry (IHC) staining and enzyme-linked immunosorbent assay (ELISA), respectively. The number of biotinylated IgG-positive cells (N = 5/group) and serum MMP9 level (N = 8/group) were lower in the TFP5 group than in vehicle group (p < 0.05), which suggested that TFP5 protected the BBB during ischemia (Fig. 5C,D; Fig. 6).

Finally, an inflammatory reaction during acute ischemic stroke could be reflected by activated microglial cells, which were marked with the ionized calcium-binding adapter molecule 1 (Iba1). IHC staining showed that the number of Iba1-positive cells were lower in the TFP5 group than in the vehicle group (p < 0.05), which suggested that injection with TFP5 after ischemia induced an anti-neuroinflammatory effect (N = 5/group; Fig. 5E,F).

## Discussion

During normal development and function of the nervous system, CDK5 and its main cofactor p35 maintain normal CDK5 activity, which is involved in a wide variety of physiological processes, including neuronal migration, synaptic activity, as well as axon and dendrite development^19^,^20^,^21^. The pathological states of neurological disorders and neurodegenerative diseases, including AD, amyotrophic lateral sclerosis, Niemann-Pick type C disease, and ischemic stroke, lead to cleavage of p35 into the more stable p25 by the calcium-dependent protease calpain and to increased CDK5 activity^22^. Recently, several interesting studies found that AD and PD outcomes could be improved by TFP5, which suppressed CDK5/p25 hyperactivity specifically^14^,^15^. Although a previous study showed that P5-TAT, which lacks the green fluorescent FITC that TFP5 contains, improved the outcomes of neonatal hypoxic-ischemic encephalopathy^23^, no prior studies have been done in adult ischemic stroke. Here, we used an *in vivo* model of ischemic stroke to explore the therapeutic effects of TFP5. Our results were significant because they provided clear evidence of neuroprotection induced by TFP5 in early-stage adult ischemic stroke.

CDK5 activity increases when its cofactor p35 is cleaved into p25 in various pathologies^6^,^7^,^8^. TFP5 and CDK5 inhibitory peptide (CIP) produced a selective reduction in abnormal CDK5 hyperactivity in AD mice, but the details of the conversion from p35 to p25 had not been shown^14^,^24^. Previous studies had indicated that TFP5 targeted CDK5 specifically, without affecting other CDKs^24^,^25^, and that CDK5 hyperactivity was inhibited by TFP5^14^,^15^,^24^,^25^. In this experiment, brain lysates were obtained from whole ischemic hemispheres in 2-mm-thick coronal slices because it was technically difficult to distinguish precisely between the ischemic core area and the penumbra. A previous study showed CDK5 had mostly dissociated in ischemic areas by 8 h post-ischemia induction^26^; however, GAPDH, which degraded just ~5% after 48 h of ischemia^27^, could not be used to normalize for the quantity of targeted proteins in brain lysates. Theoretically, p25 or p35 level depends largely on the volume of the non-infarction tissue, which was verified by the expression of p25, p35 and CDK5 in western blotting here. The p25/p35 ratio can be used as an index of p25 formation from p35 cleavage. An *in vitro* study of ischemic stroke showed that the p25/p35 ratio increased in a time-dependent manner^28^. We found that TFP5 administration decreased the p25/p35 ratio in the ischemic hemisphere, which was in accordance with a CIP-peptide study^29^. However, the higher expressions of p25 were founded in animals with surgeries than normal animals, which suggested increased p25 expression might be induced by stress during surgeries. Thus, the p25/p35 ratio would be fail with expressing the cleaving from p35 into p25 precisely.

Well-documented experimental evidence supports the critical involvement of NMDA receptor-mediated excitotoxicity in neuronal damage after stroke. However, several large-scale clinical trials have failed to find the expected efficacy of NMDA receptor antagonists in reducing brain injuries^30^,^31^,^32^, and the underlying reason might be the differential roles of NMDA receptor subunits in mediating excitotoxic neuronal death^33^. Previous studies showed that ischemia induced the phosphorylation of NR2A^S1232^ which was contributed by CDK5 signaling pathway, and a nonspecific CDK inhibitor roscovitine could inhibit the cell apoptosis^34^,^35^. In this study, the enhanced p-NR2A^S1232^ expression after ischemia was inhibited by TFP5, which indicated specific CDK5 inhibitor TFP5 protecting the brain cells after ischemia may be resulted from the inhibition on NR2A-mediated excitotoxicity. Hence, TFP5 might be a potential NR2A antagonist in the treatment for ischemic stroke.

Astrocytes perform many functions, including biochemical support for endothelial cells that form the BBB, provision of nutrients to nervous tissue, and a role in the repair and scarring processes of the central nervous system following traumatic injury. The presence of activated astrocytes is characteristic of neuroinflammation in chronic neurodegenerative diseases^36^. In a previous study about AD, there was a reduction in the number of GFAP-positive cells after TFP5 treatment^14^. In contrast, this study showed that the GFAP-positive cells in the TFP5 group had more normal morphologies than the GFAP-positive cells in the control groups. This indicated that TFP5 might protect astrocytes from damage in ischemia early-stage, and this may be beneficial for the protection of BBB and the recovery of damaged neurons.

In summary, our *in vivo* study employing an ischemic stroke model with TFP5 injection demonstrated that TFP5 is a promising therapeutic candidate for adult ischemic stroke. Although a benefit for ischemia-related neurological deficits was not demonstrated, the TFP5 peptide did provide clear neuroprotection in terms of reducing infarct size, alleviation of NR2A-mediated excitotoxicity, neuroinflammation and apoptosis, and the protection of astrocytes and the BBB. Additionally, because there are multiple mechanisms for ischemic stroke, co-treatments with multiple neuroprotective agents are being evaluated. Our previous study showed that combined treatment with hypothermia and neuroprotective agents, such as neuroglobulin and MK-801, provided better protection for ischemic stroke *in vitro*^37^. Currently, agents that specifically target CDK5 hyperactivity in ischemic stroke are scarce, but TFP5 has potential to serve as a CDK5-targeting agent and might also be used as an agent in co-treatments for ischemic stroke.

## Materials and Methods

### Animal protocol

Male Sprague-Dawley rats with weights ranging from 250–280 g were obtained from the Guangdong Medical Laboratory Animal Center (Guangzhou, China). Animals were housed individually in the animal facility. All procedures performed on rats were approved and carried out in accordance with the Institutional Animal Care and Use Committee of the Laboratory Animals Center, Nanfang Hospital, Southern Medical University. Randomized animals were used in sham surgeries, ischemic models, or post-ischemia treatment studies. For the surgical procedures, anesthesia was induced with 4% isoflurane in an induction chamber and anesthesia was maintained with 2% isoflurane delivered through a face mask (RWD, Shenzhen, China). A heating pad and bubble wrap were used to maintain each rat’s the core temperature at 37 °C throughout the surgical procedure.

### Cerebral ischemia model

A modified intraluminal filament model was used to induce MCAO as previously described^38^,^39^. After 2 h of MCAO, reperfusion was established by retracting the filament. To verify the success of MCAO surgery and reperfusion, blood flow in the cortex supplied by the MCA was measured in the core MCA territory (2 mm posterior and 6 mm lateral to bregma) by Laser Doppler Flowmetry (RWD, Shenzhen, China). The rats that did not show a reduction in blood flow below 30% of the baseline value after filament insertion were eliminated from the study. The success of reperfusion was determined by an increase in blood flow above 80% of the baseline value after retraction of the filament. During the sham surgeries, a nylon filament was inserted into the internal carotid artery approximately 5 mm from the recurved external carotid artery, and this procedure failed to induce brain ischemia.

### TFP5 design and administration

TFP5 is a 24-aa residue peptide (Lys254-Ala277) derived from truncated p35, which was described previously^14^. As a control, the Scb peptide was used (sequence below). Both TFP5 and Scb were commercially synthesized by Chinapeptides (Shanghai, China) and were dissolved in saline at a concentration of 7.5 mg/ml. The ischemic animals were immediately administered a single dose of 30 mg/kg TFP5 or Scb by intraperitoneal (i.p.) injection when reperfusion was started by retracting the filament. TFP5 or Scb was replaced with the same volume of saline in vehicle animals. Peptide sequences were as follows:

TFP5 peptide, FITCGGGKEAFWDRCLSVINLMSSKMLQINAYARAARRAARR;

Scb peptide, FITCGGGGGGFWDRCLSGKGKMSSKGGGINAYARAARRAARR.

### Infarct size analysis

After 48 h of reperfusion, the animals were killed by an overdose of chloralic hydras through the enterocoelia. The brains were removed and coronally sectioned (thickness, 2 mm) in a special groove (RWD, Shenzhen, China). The sections were immediately immersed in 4 ml of 1% TTC and incubated at 37 °C for 10 min. Afterwards, the TTC solution was replaced with 4% paraformaldehyde. The sections were photographed with a digital camera after 2 h, and the infarct size was measured by ImageJ software (National Institutes of Health, US). To eliminate the contribution of post-ischemic edema to the volume of injury, infarct size was corrected as described previously^38^,^39^. The infarct size (%) was calculated as [(volume of the left hemisphere - noninfarct volume of the right hemisphere)/volume of the left hemisphere] × 100%.

### ELISA

Serum samples were collected from animals after 48 h of reperfusion and were analyzed in duplicate for serum concentrations of MMP9 using commercially available ELISA test kits (CUSABIO, Wuhan, China). The assays were performed in accordance with the manufacturer’s specifications. Researchers who were blind to the group designations of the specimens collected all of the data.

### Western blot analyses

For analyses of p25/p35, CDK5, NR2A and p-NR2A^S1232^ expression, 2-mm-thick coronal slices 3.30 mm posterior to bregma or cortex neighbouring ischemic regions and hippocampus of ischemic hemisphere were obtained and analyzed by western blot. Briefly, tissue homogenates were prepared from animals that underwent sham surgeries or were injected with saline, TFP5, or Scb after 2 h of MCAO followed by 48 h of reperfusion. Equal amounts of total protein were resolved by SDS-PAGE and transferred to nitrocellulose membranes. Membranes were probed with primary antibodies, washed, and incubated with HRP-conjugated secondary antibodies. Membranes were exposed to X-ray film and resulting autoradiograms were scanned and analyzed using Image-Pro Plus 6.0 (Media Cybernetics Inc., US). Quantitation was presented as the normalized ratio of the optical density (OD) of the targeted protein to the OD of GAPDH. Anti-p25/p35 and anti-CDK5 were obtained from Cell Signaling Technology Inc. and Abcam Inc., and the primary antibodies were used at a 1:1000 dilution. Anti-NR2A and anti-p-NR2A^S1232^ were obtained from BIOSS (Beijing, China), and the primary antibodies were used at a 1:300 dilution.

### TUNEL and IHC staining

After 48 h of reperfusion, the rats were perfused transcardially with 4 °C saline followed by 4% paraformaldehyde. Then, the brains and major organs were removed and soaked in 4% paraformaldehyde for ≥24 h. Paraffin-embedded brains were dissected into 7 μm sections at 3 coronal levels: +1.70 mm (frontal cortex), −0.26 mm (optic chiasm), and −3.30 mm (middle of the hippocampus) from bregma. Two sections from each of the levels were stained. TUNEL staining was performed according to the manufacturer’s instructions with the TUNEL FITC Apoptosis Detection Kit (Vazyme, Nanjing, China).

IHC of GFAP, Iba1 and biotinylated IgG were performed. Briefly, 7 μm sections of the same coronal level were prepared from paraffin-embedded brains. The sections were placed on slides and incubated with primary antibodies overnight. The slides were washed with PBS and incubated with secondary antibodies before being dehydrated and coverslipped in DPX. Anti-GFAP, anti-Iba1 and anti-biotinylated IgG antibodies were obtained from Biosynthesis Biotechnology (Beijing, China), Abcam Inc. and ZSGB-BIO (Beijing, China), respectively. Images were captured using an Olympus microscope and analyzed with ImageJ software by a researcher who was blind to the group designations.

### Statistical analysis

Statistical analysis was performed with the SPSS 16.0 for Windows software package (SPSS, US). Data were expressed as means ± standard errors (SEs). One-way ANOVA was used to determine statistical differences in all of the observed indicators from the different groups. The Least Significant Difference (LSD) t-test or Dunnett T3 test, if the variance was heterogenous, was used to further analyze differences. Statistical significance was defined as p < 0.05. Numbers of animals were indicated as N in the results.

## Additional Information

**How to cite this article**: Ji, Y.-B. *et al*. TFP5 peptide, derived from CDK5-activating cofactor p35, provides neuroprotection in early-stage of adult ischemic stroke. *Sci. Rep.* **7**, 40013; doi: 10.1038/srep40013 (2017).

**Publisher's note:** Springer Nature remains neutral with regard to jurisdictional claims in published maps and institutional affiliations.

## References

[b1] Doyle K. P., Simon R. P. & Stenzel-Poore M. P. Mechanisms of ischemic brain damage. Neuropharmacology 55(3), 310–318 (2008). 18308346 10.1016/j.neuropharm.2008.01.005 PMC2603601

[b2] Knockaert M., Greengard P. & Meijer L. Pharmacological inhibitors of cyclin-dependent kinases. Trends Pharmacol Sci 23, 417–425 (2002). 12237154 10.1016/s0165-6147(02)02071-0

[b3] Rashidian J., Iyirhiaro G. & Park D. S. Cell cycle machinery and stroke. Biochem Biophys Acta 1772, 484–493 (2007). 17241774 10.1016/j.bbadis.2006.11.009

[b4] Timsit S. & Menn B. Cerebral ischemia, cell cycle elements and Cdk5. Biotechnol J 2, 958–966 (2007). 17619233 10.1002/biot.200700072

[b5] Dhavan R. & Tsai L. H. A decade of CDK5. Nat Rev Mol Cell Biol 2, 749–759 (2001). 11584302 10.1038/35096019

[b6] Bu B., Li J., Davies P. & Vincent I. Deregulation of cdk5 hyperphosphorylation, and cytoskeletal pathology in the Niemann-Pick type C murine model. J Neurosci 22, 6515–6525 (2002). 12151531 10.1523/JNEUROSCI.22-15-06515.2002 PMC6758154

[b7] Lau L. F., Seymour P. A., Sanner M. A. & Schachter J. B. Cdk5 as a drug target for the treatment of Alzheimer’s disease. J Mol Neurosci 19, 267–273 (2002). 12540052 10.1385/JMN:19:3:267

[b8] Cruz J. C., Tseng H. C., Goldman J. A., Shih H. & Tsai L. H. Aberrant Cdk5 activation by p25 triggers pathological events leading to neurodegeneration and neurofibrillary tangles. Neuron 40, 471–483 (2003). 14642273 10.1016/s0896-6273(03)00627-5

[b9] Cheung Z. H., Fu A. K. & Ip N. Y. Synaptic roles of Cdk5: implications in higher cognitive functions and neurodegenerative diseases. Neuron 50, 13–18 (2006). 16600851 10.1016/j.neuron.2006.02.024

[b10] Helal C. J. . Discovery and SAR of 2-aminothiazole inhibitors of cyclin-dependent kinase5/p25 as a potential treatment for Alzheimer’s disease. Bioorg Med ChemLett 14, 5521–5525 (2004). 10.1016/j.bmcl.2004.09.006 15482916

[b11] Menn B. . Delayed Treatment with Systemic (S)-Roscovitine Provides Neuroprotection and Inhibits *In Vivo* CDK5 Activity Increase in Animal Stroke Models. PLoS ONE 5(8), e12117 (2010). 20711428 10.1371/journal.pone.0012117 PMC2920814

[b12] Helal C. J. . Potent andcellularly active 4-aminoimidazole inhibitors of cyclin-dependent kinase 5/p25 for the treatment of Alzheimer’s disease. Bioorg Med Chem Lett 19, 5703–5707 (2009). 19700321 10.1016/j.bmcl.2009.08.019

[b13] Knockaert M. . Intracellular targets of paullones. Identification following affinity purification on immobilized inhibitor. J Biol Chem 277, 25493–25501 (2002). 11964410 10.1074/jbc.M202651200

[b14] Shukla V. . A truncated peptide from p35, a Cdk5 activator, prevents Alzheimer’s disease phenotypes in model mice. Faseb J 27, 174–186 (2013). 23038754 10.1096/fj.12-217497 PMC3528323

[b15] Binukumar B. . Peptide TFP5/TP5 derived from Cdk5 activator P35 provides neuroprotection in the MPTP model of Parkinson’sdisease. Mol Biol Cell 26(24), 4478–4491 (2015). 26399293 10.1091/mbc.E15-06-0415 PMC4666141

[b16] Akiguchi I., Tomimoto H., Suenaga T., Wakita H. & Budka H. Blood-brain barrier dysfunction in Binswanger’s disease; an immunohistochemical study. Acta Neuropathol 95(1), 78–84 (1998). 9452825 10.1007/s004010050768

[b17] Horstmann S. . Sonographic monitoring of mass effect in stroke patients treated with hypothermia. Correlation with intracranial pressure and matrix metalloproteinase 2 and 9 expression. J Neurol Sci 276, 75–78 (2009). 18834996 10.1016/j.jns.2008.08.038

[b18] Kelly P. J. . Oxidative stress and matrix metalloproteinase-9 in acute ischemic stroke: the Biomarker Evaluation for Antioxidant Therapies in Stroke (BEAT-Stroke) study. Stroke 39, 100–104 (2008). 18063832 10.1161/STROKEAHA.107.488189

[b19] Bibb J. A. . Phosphorylation of DARPP-32 by Cdk5 modulates dopamine signalling in neurons. Nature 402, 669–671 (1999). 10604473 10.1038/45251

[b20] Ohshima T. . Migration defects of cdk5(−/−)neurons in the developing cerebellum is cell autonomous. J Neurosci 19, 6017–6026 (1999). 10407039 10.1523/JNEUROSCI.19-14-06017.1999 PMC6783065

[b21] Rosales J. L., Nodwell M. J., Johnston R. N. & Lee K. Y. Cdk5/p25(nck5a) interaction with synaptic proteins in bovine brain. J Cell Biochem 78, 151–159 (2000). 10797574 10.1002/(sici)1097-4644(20000701)78:1<151::aid-jcb14>3.0.co;2-l

[b22] Su S. C. & Tsai L. H. Cyclin-Dependent Kinasesin Brain Development and Disease. Annu Rev Cell Dev Biol 27, 465–491 (2011). 21740229 10.1146/annurev-cellbio-092910-154023

[b23] Tan X. . The inhibition of Cdk5 activity after hypoxia/ischemia injury reduces infarct size and promotes functional recovery in neonatal rats. Neuroscience 290, 552–560 (2015). 25665755 10.1016/j.neuroscience.2015.01.054

[b24] Zheng Y. L. . A Cdk5 inhibitory peptide reduces tau hyperphosphorylation and apoptosis in neurons. EMBO J 24, 209–220 (2005). 15592431 10.1038/sj.emboj.7600441 PMC544899

[b25] Zheng Y. L. . A 24-residue peptide (p5), derived from p35, the Cdk5 neuronal activator, specifically inhibits Cdk5-p25 hyperactivity and tau hyperphosphorylation. J Biol Chem 285, 34202–34212 (2010). 20720012 10.1074/jbc.M110.134643 PMC2962518

[b26] Hayashi T., Warita H., Abe K. & Itoyama Y. Expression of cyclin-dependent kinase 5 and its activator p35 in rat brain after middle cerebral artery occlusion. Neuroscience Letters 265, 37–40 (1999). 10327200 10.1016/s0304-3940(99)00201-3

[b27] Ren G. M., Wang Y. Y. & Liu J. The relationship between GAPDH mRNA multiple location degradation and advanced stage postmortem intervals. J Sichuan Univ(Med Sci Edi) 40(5), 848–852 (2009). 19950597

[b28] Meyer D. A. . Ischemic stroke injury is mediated by aberrant Cdk5. The Journal of Neuroscience 34(24), 8259–8267 • 8259 (2014). 24920629 10.1523/JNEUROSCI.4368-13.2014 PMC4051977

[b29] Bosutti A. . Targeting p35/Cdk5 signalling via CIP-Peptide promotes angiogenesis in hypoxia. PLoS ONE 8(9), e75538 (2013). 24098701 10.1371/journal.pone.0075538 PMC3787057

[b30] Lee J. M., Zipfel G. J. & Choi D. W. The changing landscape of ischaemic brain injury mechanisms. Nature 399, A7–A14 (1999). 10392575 10.1038/399a007

[b31] Ikonomidou C. & Turski L. Why did NMDA receptor antagonists fail clinical trials for stroke and traumatic brain injury? Lancet Neurol 1, 383–386 (2002). 12849400 10.1016/s1474-4422(02)00164-3

[b32] Kemp J. A. & McKernan R. M. NMDA receptor pathways as drug targets. Nat Neurosci 5 [Suppl], 1039–1042 (2002). 12403981 10.1038/nn936

[b33] Liu Y. . NMDA receptor subunits have differential roles in mediating excitotoxic neuronal death both *in vitro* and *in vivo*. J Neurosci 14; 27(11), 2846–2857 (2007). 10.1523/JNEUROSCI.0116-07.2007 PMC6672582 17360906

[b34] Wang J., Liu S., Fu Y., Wang J. H. & Lu Y. Cdk5 activation induces hippocampal CA1 cell death by directly phosphorylating NMDA receptors. Nat Neurosci 6(10), 1039–1047 (2003). 14502288 10.1038/nn1119

[b35] Miao Y. . Involvement of calpain/p35-p25/Cdk5/NMDAR signaling pathway in glutamate-induced neurotoxicity in cultured rat retinal neurons. PLoS One 7(8), e42318 (2012). 22870316 10.1371/journal.pone.0042318 PMC3411656

[b36] Akiyama H. . Inflammation and Alzheimer’s disease. Neurobiol Aging 21, 383–421 (2000). 10858586 10.1016/s0197-4580(00)00124-x PMC3887148

[b37] Gao X. Y. . Combination of mild hypothermia with neuroprotectants has greater neuroprotective effects during oxygen-glucose deprivation and reoxygenation-mediated neuronal injury. Sci Rep 4, 7091 (2014). 25404538 10.1038/srep07091 PMC4665348

[b38] Ji Y. B. . Therapeutic time window of hypothermia is broader than cerebral artery flushing in carotid saline infusion after transient focal ischemic stroke in rats. Neurological Research 34(7), 657–663 (2012). 22709718 10.1179/1743132812Y.0000000061

[b39] Ji Y. B. . Intermittent intracarotid artery cold saline infusion as alternative method for neuroprotection after ischemic stroke. Neurosurgical Focus 33(1), E10 (2012). 10.3171/2012.5.FOCUS1215 22746227

